# Object Detection in Very High-Resolution Aerial Images Using One-Stage Densely Connected Feature Pyramid Network

**DOI:** 10.3390/s18103341

**Published:** 2018-10-06

**Authors:** Hilal Tayara, Kil To Chong

**Affiliations:** 1 Department of Electronics and Information Engineering, Chonbuk National University, Jeonju 54896, Korea; hilaltayara@jbnu.ac.kr; 2 Advanced Electronics and Information Research Center, Chonbuk National University, Jeonju 54896, Korea

**Keywords:** Aerial images, convolution neural network (CNN), deep learning, feature pyramid network, focal loss, object detection

## Abstract

Object detection in very high-resolution (VHR) aerial images is an essential step for a wide range of applications such as military applications, urban planning, and environmental management. Still, it is a challenging task due to the different scales and appearances of the objects. On the other hand, object detection task in VHR aerial images has improved remarkably in recent years due to the achieved advances in convolution neural networks (CNN). Most of the proposed methods depend on a two-stage approach, namely: a region proposal stage and a classification stage such as Faster R-CNN. Even though two-stage approaches outperform the traditional methods, their optimization is not easy and they are not suitable for real-time applications. In this paper, a uniform one-stage model for object detection in VHR aerial images has been proposed. In order to tackle the challenge of different scales, a densely connected feature pyramid network has been proposed by which high-level multi-scale semantic feature maps with high-quality information are prepared for object detection. This work has been evaluated on two publicly available datasets and outperformed the current state-of-the-art results on both in terms of mean average precision (mAP) and computation time.

## 1. Introduction

Object detection in very high-resolution (VHR) aerial images is a challenging task. However, it is important for a wide range of applications such as military applications [1,2], urban planning [3], and environmental management [4]. Therefore, it has attracted the attention of researchers in recent years and is considered as an essential step for understanding and interpreting large aerial scenes [5]. Thus, researchers have developed different methods and algorithms in order to detect different types of targets in VHR aerial images such as vehicle [6,7,8,9,10], airplane [11,12,13], buildings [14,15], and storage tanks [16,17].

The works that have been proposed in the literature for solving object detection task in VHR aerial images can be classified into two main categories: traditional approaches that rely on handcrafted features and deep learning-based approaches that rely on a convolution neural network (CNN) as feature extractor and provide superior performance. Handcrafted features limit the representation capacity and do not give the desired accuracy [18]. On the other hand, deep learning shows an outstanding performance in many domains such as image processing [19,20,21,22,23] due to automatic features generation.

**Figure 1:**
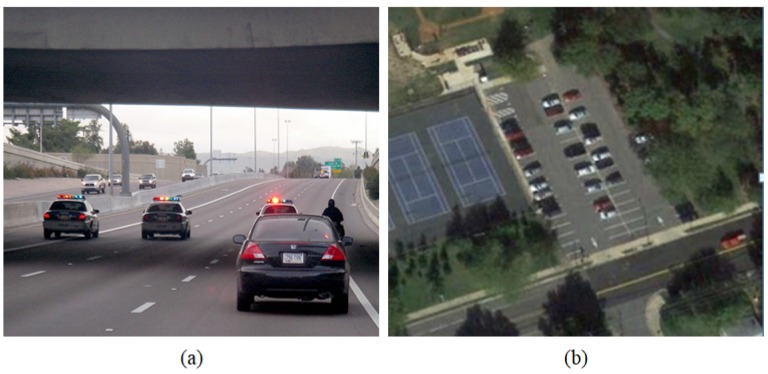
Comparison between the scales of the objects in natural images given by COCO dataset (**a**) and the scale of the objects in VHR aerial images given by NWPU VHR-10 dataset (**b**). It can be seen that the vehicles in natural images occupy a larger area compared with the vehicles in VHR aerial images.

Region-based CNNs have outperformed conventional object detection methods [21,22,24,25] in many benchmarks such as PASCAL [26] and COCO [27]. However, object detection in these benchmarks is easier than VHR aerial images benchmarks. Objects in natural images are much larger than those in the aerial images. In addition, aerial image datasets contain objects with fixed and variable shapes and scales such as ships, airplanes, and vehicles for fixed shapes and bridges and harbors for variable shapes and scales. Furthermore, the visual appearance of objects in VHR aerial images varies largely due to occlusion, shadow, illumination, resolution and viewpoint variation. Therefore, object detection in VHR aerial images is challenging and more difficult than its counterpart in natural images. Figure 1 shows an example of an image from COCO dataset [27] and Northwestern Polytechnical University very-high-resolution 10-class (NWPU VHR-10) dataset [28,29]. It can be seen that the objects in COCO dataset occupy a larger area compared to those in NWPU VHR-10 dataset.

Most of the proposed object detection methods in VHR aerial images using deep learning have relied on a two-stage Faster R-CNN [30,31]. Faster R-CNN, in the first stage, generates a predefined number of proposals that are more likely to have foreground objects using region proposal network (RPN). Then, the proposed objects are classified using a CNN. These stages should be optimized independently and the overall system is very slow. In addition, Faster R-CNN does not perform well on small-sized objects because it utilizes the last feature map of the backbone model as an input to the RPN. Therefore, works such as [31] have tried to integrate feature maps from earlier stages of the backbone network. However, the overall performance is still not satisfying and the computation time is long.

**Figure 2:**
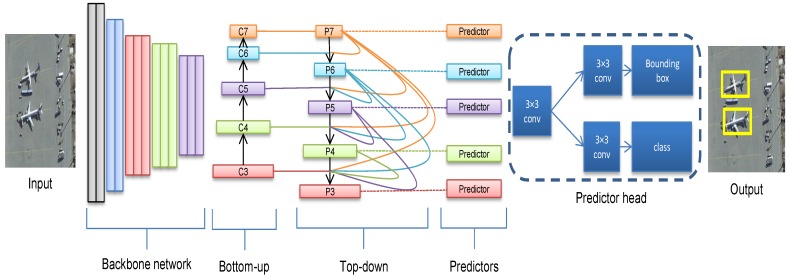
The overall architecture of the proposed model.

In this paper, a one-stage end-to-end object detection model in VHR aerial images and a densely connected feature pyramid network have been proposed. It provides high-level multi-scale semantic feature maps with high-quality information for object detection task with multi-scale appearance. Extensive experiments were carried out using different backbones such as VGG-16 [32], Resnet-50 and Resnet-101 [33]. The proposed model outperforms the state-of-the-art models introduced in the literature in terms of mean average precision (mAP) and computation time on two publicly available VHR aerial images benchmarks. Generally, the proposed model consists of four distinctive parts. The first part is the backbone network, which is the convolutional blocks of either VGG-16, Resnet 50, or Resnet 101. The second part is the bottom-up pathway which uses the last layer of the convolutional blocks of the backbone network. The third part is the top-down pathway which is the proposed densely connected feature pyramid network. The last part is the predictor head by which the classes and bounding boxes are predicted. A general overview of the proposed model is shown in Figure 2. A detailed explanation of the proposed model is given in Section 3.

The rest of the paper is organized as follows: Section 2 lists the related works published recently in the literature. Section 3 describes the methodology and implementation details. Section 4 presents datasets used for evaluating the proposed model, evaluation metrics, and experimental results. Section 5 concludes the paper.

## 2. Related Works

Over the past years, object detection in VHR aerial images has been extensively studied. It requires learning classifiers that are able to discriminate between the foreground and background objects in the given image. Hence, the input of the classifiers is the extracted features by either sliding windows or object proposal. Therefore, feature extraction is an essential step in developing successful object detection systems. Different approaches have been proposed for low-level feature extraction, such as local binary pattern (LBP), histogram of oriented gradients (HOG), sparse coding, and bag of words (BoW). Currently, on the other hand, deep learning approaches are widely used due to the powerful feature extraction and performance improvement of object detection task. For instance, AlexNet [23] was first used for VHR aerial images and outperformed Fisher discrimination dictionary learning (FDDL) [34], spatial sparse coding BoW (SSCBoW) [35], BoW [36], and the collection of part detectors (COPD) [37]. CNN-based object detection models can be categorized into two groups, namely region-based CNN models such as R-CNN [38], Fast R-CNN [21] and Faster R-CNN [22] and uniform models that are region free such as You Only Look Once (YOLO) [25] and its variants, single shot multibox detector (SSD) [24] and Retinanet [39]. Region-based CNN utilized a selective search algorithm for extracting around 2000 object proposals. Then, the features of the proposed objects are extracted using a pre-trained CNN and classified using a linear support vector machine (SVM) [38]. The performance of R-CNN outperformed handcrafted feature-based methods. Therefore, Fast R-CNN was proposed in order to increase detection accuracy and decrease computation time. They used the region of interest (RoI) and fully connected layers for classifying the objects proposed. RPN was added to Fast R-CNN in order to propose high-quality regions. This network was called Faster R-CNN and outperformed the ancestor models with a higher speed [22]. On the other hand, uniform one-stage models such as YOLO [25], SSD [24], and Retinanet [39] solved object detection task using regression by which a one-stage network predicts bounding boxes and their classes. YOLO model was faster than the all other CNN-based object detection models. SSD applied small convolution filters to feature maps instead of using fully connected layer such as YOLO. In addition, SSD makes predictions using feature maps at different scales which in turn increased the mAP. Recently, Retinanet was proposed by [39]. They introduced focal loss function in order to deal with data imbalance occurred by the plenty of background objects. Rotation-invariant CNN model was introduced by [29]. They improved the performance of object detection by adding a new rotation-invariant layer to an existing CNN. Tang et al. [31] proposed using hyper-region proposal network (HRPN) and boosted classifiers to detect vehicles in the VHR aerial images. Markov random field was combined with CNN in the work proposed by Yang et al. [40]. Semisupervised learning was utilized in different works in order to solve object detection in VHR aerial images [41,42]. An iterative weakly supervised learning model was proposed by Zhang et al. [2], by which they extracted the proposals and located the aircraft in VHR aerial images. R-CNN was used in [43] for oriented building detection in satellite images. The performance of object detection in VHR aerial images has been improved by using semantic segmentation model [44] and Faster R-CNN [45]. Xu et al. in [46] introduced an end-to-end deformable CNN for object detection in VHR aerial images. A multi-scale CNN was proposed by Wei et al. in [47], by which they used feature pyramid network for multi-scale object detection in VHR aerial images. Ke et al. in [48] proposed a rotation-insensitive and context-augmented object detection model in VHR aerial images.

## 3. Methodology

This section introduces the proposed model, the loss functions, and the implementation details.

### 3.1. The Proposed Model

**Figure 3:**
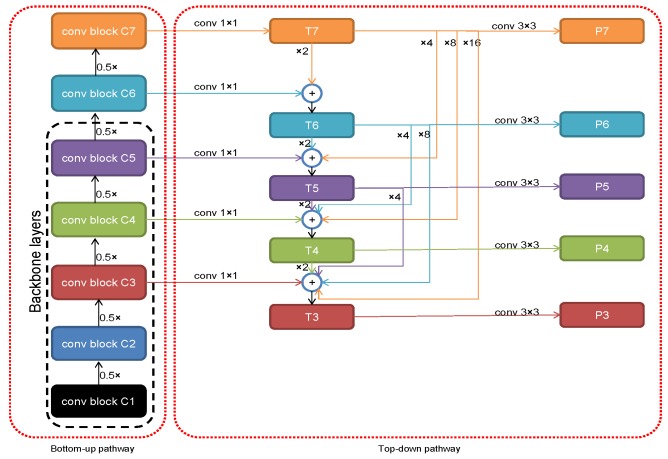
The architecture of the densely connected feature pyramid network.

The overall framework of our proposed model is depicted in Figure 2. It consists of four components namely backbone, bottom-up pathway, top-down pathway, and classification and regression heads. In this paper, VGG-16 [32], Resnet-50 and Resnet-101 [33] have been tested as the backbone in our experiments. These backbones, in general, consist of five convolution blocks. In order to build the bottom-up pathway, we select from the backbone the last convolution layer of the convolution block 3, convolution block 4, and convolution block 5 as {C3, C4, and C5}, respectively. Then, we add the feature maps C6, and C7 for having more refined semantic information. Feature maps C6 and C7 are calculated as follows:

(1) C6=Conv2D(k=256,s=(3,3),d=(2,2))(C5)

(2) C7=Conv2D(k=256,s=(3,3),d=(2,2))(ReLU(C6))

where Conv2D is a two-dimensional convolution operator which convolves a given feature map with a predefined number of kernels, *k* is the number of the kernels, *s* represents the sizes of the kernel, *d* is the strides on vertical and horizontal directions, and ReLU is the rectified linear unit activation function. Thus, the feature map C6 is carried out by convolving the feature map C5 with 256 kernels with kernel sizes equal to (3, 3) and strides equal to (2, 2) on vertical and horizontal directions. The feature map C7 is calculated by first applying ReLU activation function on the feature map C6 then convolving the resultant output by 256 kernels with kernel sizes equal to (3, 3) and strides equal to (2, 2) on vertical and horizontal directions. Thus, the bottom-up pathway produces feature maps {C3, C4, C5, C6, and C7} where the strides are {8, 16, 32, 64, and 128} for each feature map, respectively. Top-down pathway is obtained by constructing densely connected feature pyramid network {P3, P4, P5, P6, and P7}. These maps are calculated as follows:

(3) $$R_{N}=Conv2D\left(k=256,s=\left(1,1\right),d=\left(1,1\right)\right)\left(C_{N}\right)$$

(4) $$T_{7}=R_{7}$$

(5) $$T_{N}=R_{N}+\sum_{i=N+1}^{7}Up\_Sample\_Like\left(T_{i},C_{N}\right)$$

(6) $$P_{N}=Conv2D\left(k=256,s=\left(3,3\right),d=\left(1,1\right)\right)\left(T_{N}\right)$$

for N=3,4,5,6,7 in (3) and (6), and N=3,4,5,6 in (5) where $R_{N}$ is used for dimension reduction by convolving each map from the bottom-up pathway with 256 kernels with kernel sizes and strides equal to (1,1). $T_{N}$ represents densely connected feature map. $Up\_Sample\_Like(T_{i},C_{N})$ operator resizes $T_{i}$ to the size of the $C_{N}$. $P_{N}$ is the output feature map of the top-down pathway and has 256 channels. Figure 3 shows the detailed calculation of the top-down densely connected feature pyramid pathway.

**Figure 4:**
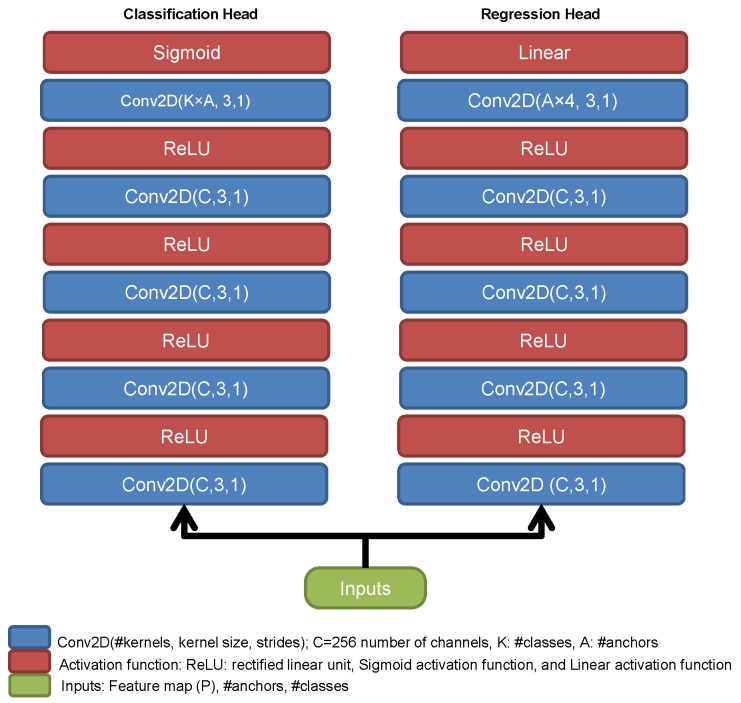
The architecture of classification and regression heads.

Each point in the feature maps of the densely connected feature pyramid network generates 9 anchors and each feature map has its own classification and regression heads. Figure 4 shows the detailed architecture of classification and regression heads. They consist of four 3 × 3 two-dimensional convolutions followed by the ReLU activation function. However, the last convolution layer in the classification head has #anchors×#classes channels followed by the sigmoid activation function and the last convolution layer in the regression head has #anchors×4 channels followed by a linear activation function. The relative offset between the ground-truth and the anchor is calculated based on [38,39]. The weights of the classification and regression heads are shared among the feature maps of the densely connected feature pyramid network. Unlike two-stage detectors that propose 2*k* boxes after non-maximum suppression, one-stage detectors propose 10*k* to 100*k* boxes per image. Therefore, more background boxes are proposed which in turn leads to data imbalance problem. In order to remedy this problem, there are two approaches in machine learning: oversampling/downsampling the minority/majority classes, or modifying the weights in the loss function. The first approach is applied in works such as Faster R-CNN and SSD. In this paper, the second approach has been followed by changing the weights in the loss function. Focal loss function that was proposed by [39] has been utilized. It modifies the cross-entropy loss in a way it that down-weights the loss assigned to easy and well-classified examples and concentrates the training on difficult ones.

### 3.2. Loss Function

Loss function is combined of bounding box regression and classification loss functions.

#### 3.2.1. Bounding Box Regression Loss Function

The relative offset between the ground-truth bounding box and the corresponding anchor has been calculated based on [38,39]. Let $\left(X_{1b},Y_{1b}\right)$ and $\left(X_{2b},Y_{2b}\right)$ be the top-left and bottom-right corners of the ground-truth bounding box and let $\left(X_{1a},Y_{1a}\right)$ and $\left(X_{2a},Y_{2a}\right)$ be the top-left and bottom-right corners of the corresponding anchor. Then targets are calculated as follows:

(7) $$\begin{array}{cc} W_{a} & =X_{2a}-X_{1a} \end{array}$$

(8) $$\begin{array}{cc} H_{a} & =Y_{2a}-Y_{1a} \end{array}$$

(9) $$\begin{array}{cc} X_{1t} & =\left(X_{1b}-X_{1a}\right)/W_{a} \end{array}$$

(10) $$\begin{array}{cc} Y_{1t} & =\left(Y_{1b}-Y_{1a}\right)/H_{a} \end{array}$$

(11) $$\begin{array}{cc} X_{2t} & =\left(X_{2b}-X_{2a}\right)/W_{a} \end{array}$$

(12) $$\begin{array}{cc} Y_{2t} & =\left(Y_{2b}-Y_{2a}\right)/H_{a} \end{array}$$

where $W_{a}$ and $H_{a}$ are the width and the height of the anchor and $\left(X_{1t},Y_{1t}\right)$ and $\left(X_{2t},Y_{2t}\right)$ are the top-left and bottom-right corners of the targets, respectively. These targets are normalized using normal distribution with μ=0 and σ=0.2. Then Let $\left(X_{1p},Y_{1p}\right)$ and $\left(X_{2p},Y_{2P}\right)$ be the top-left and bottom-right corners of the predicted bounding box. Then regression loss is carried out using smooth L1 function as follows:

(13) $$L_{reg}\left(t_{i},p_{i}\right)=smooth_{L1}\left(t_{i}-p_{i}\right)$$

(14) $$smooth_{L1}\left(d\right)=\left\{\begin{array}{cc} 0.5d^{2}, & \mathrm{if}|d|<1\\ |d|-0.5, & \mathrm{otherwise} \end{array}\right.$$

#### 3.2.2. Classification Loss Function

Focal loss function has been utilized in order to deal with the large class imbalance since the background samples are more than the foreground ones [39]. Here the concept of focal loss function is explained briefly. Focal loss function concentrates on hard examples and down-weights easy ones by adding a fine-tuning factor ${\left(1-p_{t}\right)}^{\gamma }$ to the cross-entropy loss and using the factor α that balances the importance of negative/positive cases. $p_{t}$ is the output probability *p* of the model when the target label y=1 otherwise it is 1-p. Therefore the cross-entropy for binary classification case is $CE\left(p,y\right)=-\log\left(p_{t}\right)$. Focal loss function is defined as [39]:

(15) $$FL\left(p_{t}\right)=-\alpha_{t}{\left(1-p_{t}\right)}^{\gamma }\log\left(p_{t}\right).$$

It can be noticed that the loss function is just the cross-entropy loss in the case of misclassified examples as $p_{t}$ is small and the fine-tuning factor is near one. Well-classified examples will make $p_{t}$ approach one which in turn drives the fine-tuning factor to near zero. Thus, the loss is down-weighted for well-classified examples. The rate of down-weighting the loss is controlled by γ. In our experiments the work proposed by [39] has been followed by setting the hyper-parameters α=0.25 and γ=2.

### 3.3. Implementation Details

**Figure 5:**
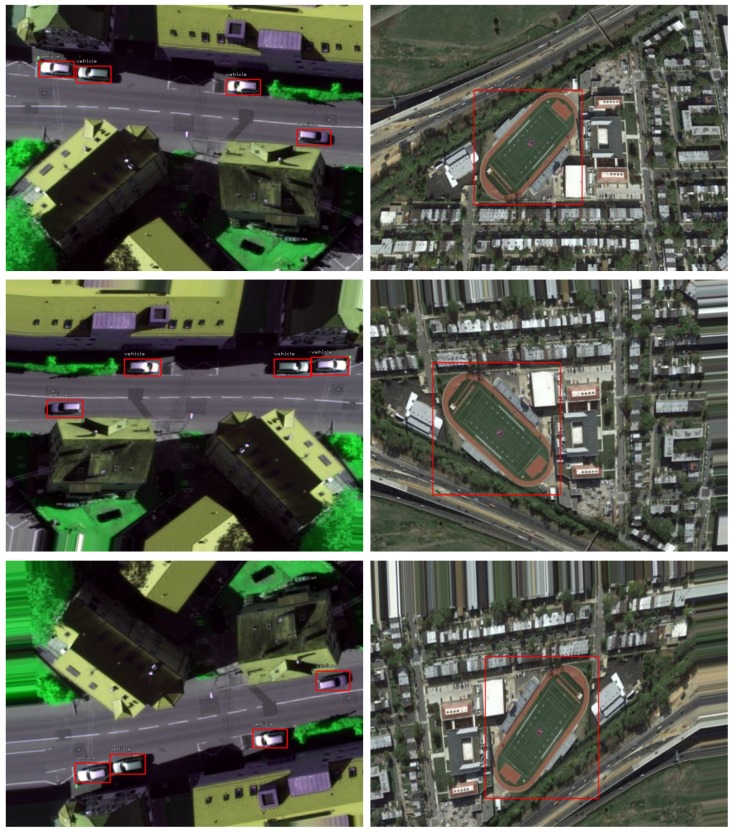
Examples of data augmentation technique. First row represents the input images while the second and third rows represent the augmented output.

Our implementation is based on a modified version of the framework introduced by [49]. This framework uses Keras and Tensorflow libraries. Data augmentation is used in order to increase training samples. Random rotation, translation, shearing, scaling, and vertical and horizontal flipping are used. Data augmentation is a process of generating artificially altered images of each instance image within training dataset. This technique results in obtaining large amount of training data, preventing over fitting, and boosting the performance of the proposed model. In addition, it is helpful in training big models with small datasets such as datasets that are used in these experiments. Generally, each input image goes under a series of transformation in order to obtain the augmented output. Figure 5 shows examples of applying augmentation on two input images. The number of epochs is set to 50 with 10,000 iterations for each epoch. The minimum and maximum lengths of the input images are set to 600 and 1000 pixels, respectively. The backbone weights are initialized using a pre-trained network on ImageNet large-scale visual recognition challenge (ILSVRC) dataset [50]. Convolution layers in the classification and regression heads are initialized using normal distribution with μ=0 and σ=0.01. The biases *b* are set to zero except the last convolution layer in the classification head is set to $b=-\log\left((1-\beta )/\beta \right)$ [39]. The parameter β is set to 0.01 at the beginning of the training and states that every anchor is labeled as foreground with a confidence of ∼β. This configuration of β prevents loss destabilization at the beginning of the training. The sizes of the anchors are set to {32, 64, 128, 256, 512} and strides to {8, 16, 32, 64, 128}. The ratios of the anchors for each anchor size are {0.5, 1, 2}. Adam optimizer is used for the optimization.

## 4. Experimental Results

In this section, Dataset description, evaluation metrics, experimental results, and comparison with the state-of-the-art models are presented.

### 4.1. Datasets Description

The proposed model has been evaluated on the widely used NWPU VHR-10 dataset [28,29]. This dataset provides 650 annotated images where each image contains at least one object. These images were annotated manually with bounding boxes as ground-truth. NWPU VHR-10 dataset is a challenging one because it contains both 565 remote sensing images with a spatial resolution (0.2 m to 2 m) and 85 pan-sharpened images with a 0.08 m spatial resolution. It has 10 different object types namely: ship, vehicle, bridge, harbor, ground track field, baseball diamond, tennis court, basketball court, storage tank, and airplane. The provided 650 images contain 302 ships, 477 vehicles, 124 bridges, 224 harbors, 163 ground track fields, 390 baseball diamonds, 524 tennis courts, 159 basketball courts, 655 storage tanks, and 757 airplanes. These details are listed in Table 1. Image sizes vary from 533×597 to 1728×1028 pixels and objects to be detected have different scales and shapes. In all experiments, dataset has been divided into 60% for training, 10% for validation, and 30% for testing. Correct detection is said to be true positive if more than 50% of the predicted bounding box overlaps with the ground-truth otherwise it is a false positive. For further evaluation, the proposed model has been tested on RSOD [51] dataset. This dataset contains 2326 images captured by Google Earth and has four classes: aircraft, overpass, oil tank, and playground.

### 4.2. Evaluation Metrics

The widely adopted precision-recall curve and average precision (AP) have been used in order to quantitatively evaluate the performance of the proposed model.

#### 4.2.1. Precision-Recall Curve

Precision represents the parts of detection that are true positives whereas recall represents the correctly identified part of positives. Precision and recall are given as:

(16) $$precision=\frac{TP}{TP+FP}$$

(17) $$recall=\frac{TP}{TP+FN}$$

where *TP*: true positive, *FN*: false negative, and *FP*: false positive. True positive case represents overlapping between the ground-truth and the predicted bounding box with more than 0.5; otherwise, it is a false positive.

#### 4.2.2. Average Precision

This metric represents the area under the precision-recall curve in the interval of recall=0 to recall=1. Higher AP means better performance and vice versa. In addition, mAP is the average value of AP over all classes and it is used for deciding the rank of the proposed models in object detection task.

### 4.3. Results

**Figure 6:**
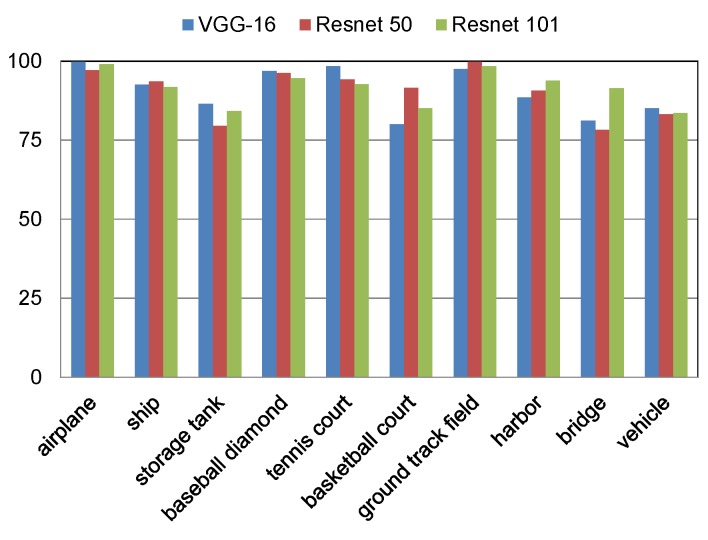
Detection results of the proposed model in terms of AP using different backbones: VGG-16, Resnet 50, and Resnet 101.

The proposed model has been tested with three different backbones namely VGG-16 [32], Resnet 50, and Resnet 101 [33]. All three backbones outperform the stated-of-the-art models. Figure 6 shows a comparison of AP for the different backbones. The achieved mAPs for VGG-16, Resnet 50, and Resnet 101 backbones are 0.9063, 0.9042, and 0.9146, respectively. In addition, the proposed model has been compared with the following methods for quantitative evaluation:

- Bag of Words (BoW) [36]: This work utilized K-mean algorithm for generating histogram of visual words by which each image region is represented.
- Spatial Sparse Coding BoW (SSCBoW) [35]: This work utilized sparse coding algorithm for generating visual words.
- The Collection of Part Detector (COPD) [37]: This method utilized 45 seed-part SVM linear detectors. They were trained on the feature extracted by HOG and resulted in a rotation-invariant object detection model.
- A transfered CNN Model [23]: This work used AlexNet network as feature extractor and achieved good results on object detection on PASCAL dataset [26].
- Rotation-invariant CNN (RICNN) [29]: This work added a new layer to Alexnet for dealing with rotated objects.
- Faster R-CNN [22]: It is a two-stage object detection CNN. The first stage proposes a set of objects whereas the second stage classifies them.
- Single Shot Multibox Detector (SSD) [24]: It is a uniform one-stage model that utilizes the feature maps at different scales.
- Rotation-insensitive CNN [48]: This work proposed context-augmented feature fusion model and RPN with multi-angle anchors.
- Deformable CNN [46]: This work proposed a deformable region-based fully convolution layer by using a deformable convolution layer instead of the conventional one.
- Multi-Scale CNN [47]: In this work, feature maps with high semantic information at different scales were proposed.

The best results in Table 2, Table 3 and Table 4 are written in bold format.

**Figure 7:**
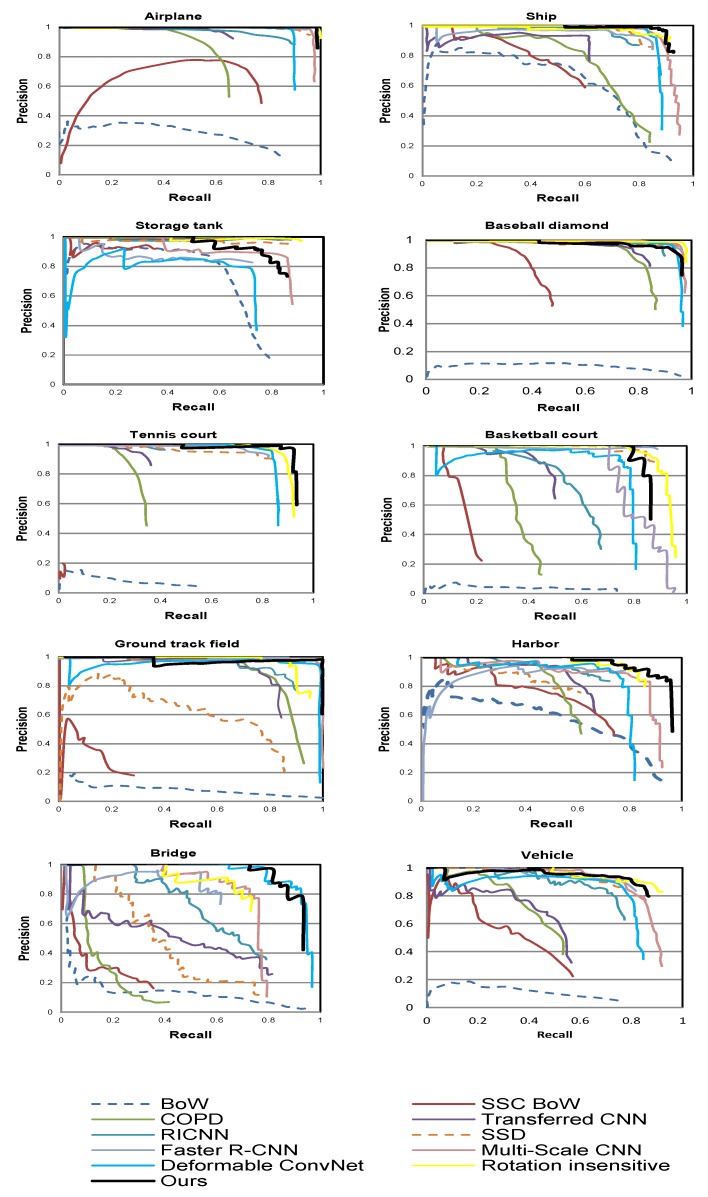
Comparison of area under precision-recall curve with different state-of-the-art models.

Table 2 shows that the proposed model outperforms the state-of-the-art models in terms of mAP with three different backbones. More specifically, the proposed model achieves 1.85%, 0.81%, and 1.02% improvement in mAP using Resnet 101, Resnet 50, and VGG-16 backbones, respectively. In addition, a remarkable improvement in some targets by using different backbones has been achieved such as 8.71% in the harbor, 19.52% in the bridge by using Resnet 101 as a backbone, and 7.59% in the tennis court by using VGG-16 as a backbone. Moreover, our proposed model outperforms the state-of-the-art models in terms of computation time. The average estimated time for processing one image is 0.088sec using Resnet 101 as a backbone. All experiments were held on a workstation with Titan X graphical processing unit which has 12 GB memory, Xeon CPU E5-2640 with 2.40GHz, and 256 GB RAM. Table 3 shows computation time comparison with the above-mentioned methods. In addition, the precision-recall curve has been studied. Figure 7 shows comparison of the precision-recall curve of the proposed model using Resnet 101 backbone with the state-of-the-art models. This metric is one of the main signs of the effectiveness and robustness. The *y*-axis represents the precision and the *x*-axis represents the recall. Better performance is indicated by the curve on the top. The results of our proposed model using Resnet 101 backbone, BoW, SSCBoW, COPD, a transferred CNN model, RICNN, SSD, faster R-CNN, rotation-insensitive CNN, multiscale CNN, and deformable CNN have been plotted.

**Figure 8:**
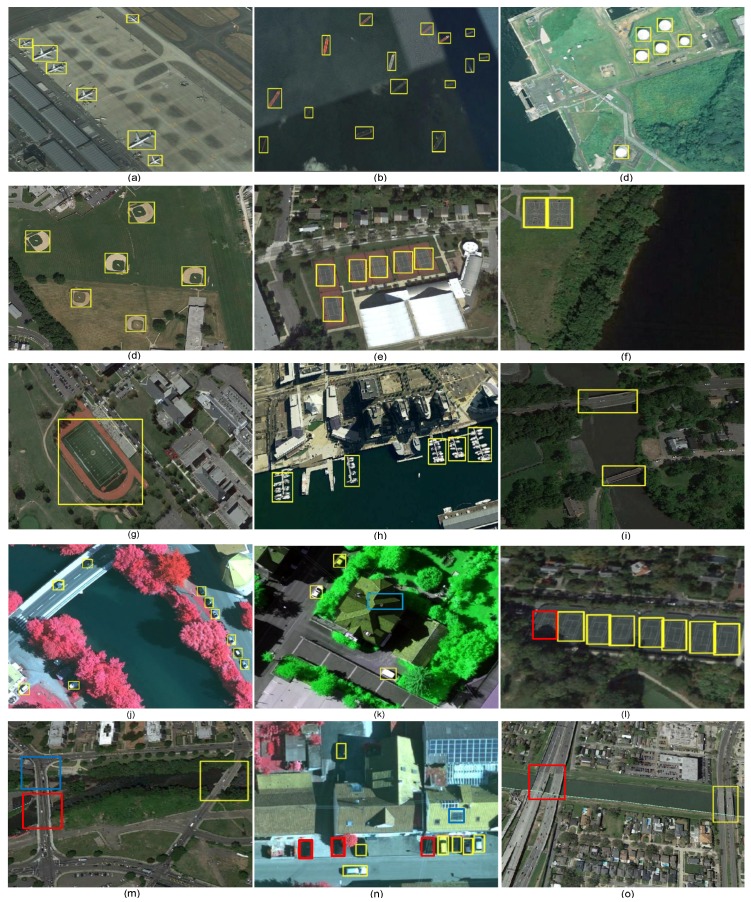
Some object detection results from NWPU VHR-10 dataset. Yellow, red, and blue colors represent true positive, false negative, and false positive cases, respectively. (**a**) airplane, (**b**) ship, (**c**) storage tank, (**d**) baseball diamond, (**e**) tennis court, (**f**) basketball court, (**g**) ground track field, (**h**) harbor, (**i**) bridge, (**j**) vehicle, (**k**–**o**) show some false positive and false negative cases.

Some of the detection results are presented in Figure 8. Yellow, red, and blues colors represent true positive, false negative, and false positive, respectively. It can be seen that the proposed model is able to detect target objects successfully regardless to their shapes, orientations, sizes, and appearances. More specifically, it can be seen that there is a big difference in terms of the size between vehicles and ground track fields and proposed model is able to deal with such difference successfully. It can be also seen that airplanes appear in different scales and the proposed model is able to detect them perfectly. In addition, the proposed model can detect objects regardless to their orientations such as ships. Some objects have similar appearance and are detected correctly such as a basketball court and tennis court.

**Figure 9:**
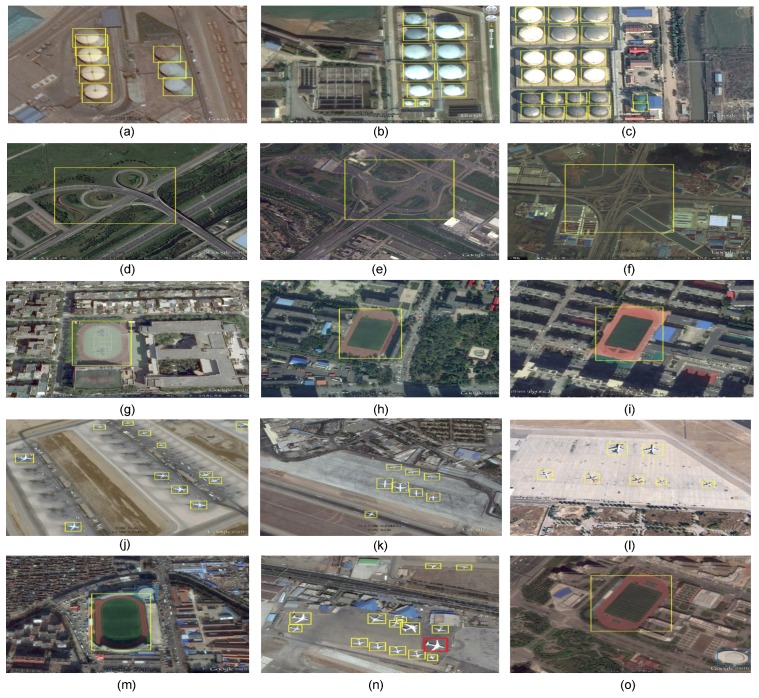
Some object detection results from RSOD dataset. Yellow, red, and blue colors represent true positive, false negative, and false positive cases, respectively. (**a**–**c**) show examples of true positive detection of oil tank, (**d**–**f**) show examples of true positive detection of overpass, (**g**–**i**) show examples of true positive detection of playground, (**j**–**l**) show examples of true positive detection of aircraft, and (**m**–**o**) show examples of false positive and false negative cases.

To further evaluate the proposed model, the proposed model has been tested on RSOD dataset [51]. Table 4 shows the comparison results of the proposed model with different versions of deformable CNN [46] and R-P-Faster R-CNN [52]. It can be seen that the proposed model outperforms the state-of-the-art models with different backbones. The oil tank class in RSOD and storage tank in NWPU VHR-10 dataset are similar, but the performance of the proposed model on RSOD outperforms the performance on NWPU VHR-10 dataset. The main reason is that only 28 images that contain storage tank are available in NWPU VHR-10 dataset. On the other hand, there are 195 images for oil tank class in RSOD dataset. Thus, the unavailability of training example is the main reason for having less accuracy in the case of storage tank. Some of the detection results from RSOD dataset are shown in Figure 9. It can be also seen that the proposed model is able to successfully detect target objects with different shapes, scales, orientations, and appearances.

## 5. Conclusions

A one-stage densely connected feature pyramid network model for object detection in VHR aerial images has been introduced. Using a densely connected pyramid network enables the model to detect target objects at different scales. This is through merging feature maps of the bottom-up pathway with the feature maps of the top-down pathway. This combination results in obtaining semantic feature maps with high-quality information at different scales. In addition, the problem of data imbalance was solved by using focal loss function. Our proposed model was tested on two publicly available benchmarks and outperformed the state-of-the-art models on both in terms of mAP and computation time.

## Figures and Tables

**Table 1:** Statistical information about NWPU VHR-10 dataset. This dataset has been divided into 60% training set, 10% validation set, and 30% testing set.

Class	# Instances
airplane	757
ship	302
storage tank	655
baseball diamonds	390
tennis courts	524
basketball court	159
ground track filed	163
harbors	224
bridge	124
vehicle	477

**Table 2:** Performance Comparison between the proposed model and the state-of-the-art models on NWPU VHR-10 dataset.

Method	Air Plane	Ship	Storage Tank	Baseball Diamond	Tennis Court	Basketball Court	Ground Track Field	Harbor	Bridge	Vehicle	mAP
BoW	0.2496	0.5849	0.6318	0.0903	0.0472	0.0322	0.0777	0.5298	0.1216	0.0914	0.2457
SSC BoW	0.5061	0.5084	0.3337	0.4349	0.0033	0.1496	0.1007	0.5833	0.1249	0.3361	0.3081
COPD	0.6225	0.6887	0.6371	0.8327	0.3208	0.3625	0.8531	0.5527	0.1479	0.4403	0.5458
Transferred CNN	0.661	0.569	0.843	0.816	0.35	0.459	0.8	0.62	0.423	0.429	0.597
RICNN	0.8835	0.7734	0.8527	0.8812	0.4083	0.5845	0.8673	0.686	0.6151	0.711	0.7263
SSD	0.957	0.829	0.856	0.966	0.821	0.86	0.582	0.548	0.419	0.756	0.7594
Faster R-CNN	0.946	0.823	0.6532	0.955	0.819	0.897	0.924	0.724	0.575	0.778	0.8094
Deformable CNN	0.873	0.814	0.636	0.904	0.816	0.741	0.903	0.753	0.714	0.755	0.7909
Rotation-Insensitive CNN	0.997	0.908	**0.9061**	0.9291	0.9029	0.8013	0.9081	0.8029	0.6853	**0.8714**	0.8712
Multi-Scale CNN	0.993	0.92	0.832	**0.972**	0.908	**0.926**	0.981	0.851	0.719	0.859	0.8961
Ours (VGG-16)	**0.9977**	0.926	0.8652	0.9689	**0.9839**	0.7997	0.9752	0.8846	0.8111	0.8514	0.9063
Ours (Resnet 50)	0.971	**0.9361**	0.7958	0.9628	0.9424	0.9149	**0.998**	0.9071	0.782	0.8315	0.9042
Ours (Resnet 101)	0.9906	0.9182	0.842	0.9459	0.9263	0.8503	0.9839	**0.9381**	**0.9142**	0.8359	**0.9146**

**Table 3:** Computation time comparison of different models.

Methods	Average Running Time per Image (s)
BoW	5.32
SSC BoW	40.32
COPD	1.07
Transferred CNN	5.24
RICNN	8.77
SSD	0.09
Faster R-CNN	0.16
Deformable CNN	0.201
Rotation-Insensitive CNN	2.89
Multi-Scale CN	0.11
Ours (Resnet 101)	**0.088**

**Table 4:** Performance Comparison between the proposed model and the state-of-the-art models on RSOD dataset.

Method	Aircraft	Oil Tank	Overpass	Playground	mAP
R-P-Faster R-CNN	0.7084	0.9019	0.7874	0.9809	0.8447
Deformable R-FCN (ResNet-101)	0.7150	0.9026	0.8148	0.9953	0.8570
Deformable R-FCN (ResNet-101) and arcNMS	0.7187	0.9035	0.8959	0.9988	0.8792
Ours (VGG-16)	**0.8764**	**0.9712**	0.9310	**1.0**	**0.9447**
Ours (Resnet 50)	0.8576	0.9555	0.8528	0.9955	0.9153
Ours (Resnet 101)	0.8625	0.9598	**0.9467**	0.9987	0.9419
